# The association of aggressive and chronic periodontitis with systemic manifestations and dental anomalies in a jordanian population: a case control study

**DOI:** 10.1186/1746-160X-6-30

**Published:** 2010-12-29

**Authors:** Khansa T Ababneh, Anas H Taha, Muna S Abbadi, Jumana A Karasneh, Yousef S Khader

**Affiliations:** 1 Division of Periodontology, Department of Preventive Dentistry, Faculty of Dentistry, Jordan University of Science and Technology, Jordan; 2 Division of Oral Medicine, Department of Oral surgery, Oral Medicine, Oral Pathology and Radiology, Faculty of Dentistry, Jordan University of Science and Technology, Jordan; 3 Community Medicine & Public Health, Faculty of Medicine, Jordan University of science and Technology, Jordan

## Abstract

****Background**:**

The relationship between dental anomalies and periodontitis has not been documented by earlier studies. Although psychological factors have been implicated in the etiopathogenesis of periodontitis, very little information has so far been published about the association of anxiety and depression with aggressive periodontitis. The aim of this study was to investigate the association of chronic periodontitis and aggressive periodontitis with certain systemic manifestations and dental anomalies.

****Methods**:**

A total of 262 patients (100 chronic periodontitis, 81 aggressive periodontitis and 81 controls), attending the Periodontology clinics at Jordan University of Science and Technology, Dental Teaching Centre) were included. All subjects had a full periodontal and radiographic examination to assess the periodontal condition and to check for the presence of any of the following dental anomalies: dens invaginatus, dens evaginatus, congenitally missing lateral incisors or peg-shaped lateral incisors. Participants were interrogated regarding the following: depressive mood, fatigue, weight loss, or loss of appetite; and their anxiety and depression status was assessed using the Hospital Anxiety and Depression (HAD) scale.

****Results**:**

Patients with aggressive periodontitis reported more systemic symptoms (51%) than the chronic periodontitis (36%) and control (30%) patients (*p *< 0.05). Aggressive periodontitis patients had a higher tendency for both anxiety and depression than chronic periodontitis and control patients. Dental anomalies were significantly (*p *< 0.05) more frequent among both of chronic and aggressive periodontitis patients (15% and 16%, respectively), compared to controls.

****Conclusion**:**

In this group of Jordanians, systemic symptoms were strongly associated with aggressive periodontitis, and dental anomalies were positively associated with both aggressive and chronic periodontitis.

## Background

Periodontitis is a multifactorial disease that involves infection and inflammation of the supporting periodontal tissues leading to their destruction [1]. This paper focuses on two types of periodontitis: chronic periodontitis (CP) and aggressive periodontitis (AP) and their association with certain dental anomalies and psychological stress. Page and colleagues in 1983 [2] have reported that rapidly progressive periodontitis (RPP, currently termed generalized AP) progresses in alternate phases of disease activity and quiescence. They reported that the active phase of RPP is associated with systemic manifestations such as depression, malaise, weight loss and loss of appetite in some individuals.

Numerous diseases of the dentition exist that may involve the crowns or roots of teeth so that the size, shape or number of teeth may be affected. Dens invaginatus is an uncommon developmental malformation that shows a wide spectrum of anatomic variations [3]. It is believed that it arises from infolding of the dental papilla or the distortion of the enamel organ during tooth development [4-6]. The reported prevalence of dens invaginatus ranges between 0.04 to 10% [7]. The most affected permanent teeth are the maxillary lateral incisors, frequently bilateral followed by central incisors, canines, premolars and molars [8]. Clinicians most commonly use the classification proposed by Oehlers (1957) [5] which classifies dens invaginatus into:

• Type I: an enamel-lined invagination within the crown and not extending beyond the cementoenamel junction (CEJ).

• Type II: the enamel invagination into the root, beyond the CEJ, ending as a blind sac.

• Type III: the extension of the enamel-lined invagination through the root to form an additional apical or lateral foramen; usually, there is no direct communication with the pulp.

Dens evaginatus or talon cusp is a relatively rare odontogenic anomaly arising during tooth morphodifferentiation [9]. The accessory cusp varies in size, shape, length and mode of attachment to crown. It ranges from an enlarged cingulum to a large, well-delineated cusp [10]. It is usually associated with the palatal aspects of the maxillary anterior teeth [11], but may also be present on the occlusal aspects of posterior teeth, especially in people of Asian origin [12].

Peg (conical)-shaped maxillary lateral incisors are relatively common dental anomalies [13-16], that may occur in healthy individuals or as part of other diseases such as Down's syndrome [17]. In their study on Jordanian dental students, Albashaireh & Khader (2006) [15] reported that the prevalence of peg-shaped lateral incisors was 2.3%.

Hypodontia, the congenital absence of teeth, has been classified into two classes: syndromic, and nonsyndromic, depending on the cause of hypodontia [18]. The upper lateral incisors and second premolars are the most frequently affected teeth [19]. A 5.5% prevalence of hypodontia has been reported in Jordan [15].

The aims of this study were to examine the association of certain systemic manifestations with both AP and CP, to assess the anxiety and depression status in both types of periodontitis using the Hospital Anxiety and Depression (HAD) scale and to explore the association of CP and AP with certain dental anomalies. To the best of our knowledge, and based on extensive Medline search, the association between AP/CP and dental anomalies such as dens invaginatus, dens evaginatus, peg-shaped and missing lateral incisors has never been reported in the literature.

## Methods

This investigation was undertaken with the understanding and consent of each participating subject and has been conducted in full accordance with ethical principles of the World Medical Association Declaration of Helsinki http://www.wma.net/en/30publications/10policies/b3/index.html. The study has been independently reviewed and approved by The Ethical Review Board, Jordan University of Science and Technology (JUST). Written consent forms for interview and examination were signed by all participants or the parents of participants under the age of 18 years. The study population of this case-control study consisted of 262 individuals and included 100 CP cases, 81 AP cases and 81 controls. There were 125 males and 137 females with an age range of 14-71 years and a mean age of 31.3 (± 11.4 SD) years. Clinical examination was performed in the Periodontology clinic, JUST Dental Teaching Centre. The study included systemically healthy individuals who have not received any periodontal treatment in the last three months prior to examination. Individuals with diabetes mellitus or blood disorders, patients on any long-term medications, pregnant women, patients with previous or ongoing orthodontic treatment and children under the age of 14 years were excluded from the study.

**Figure 1:**
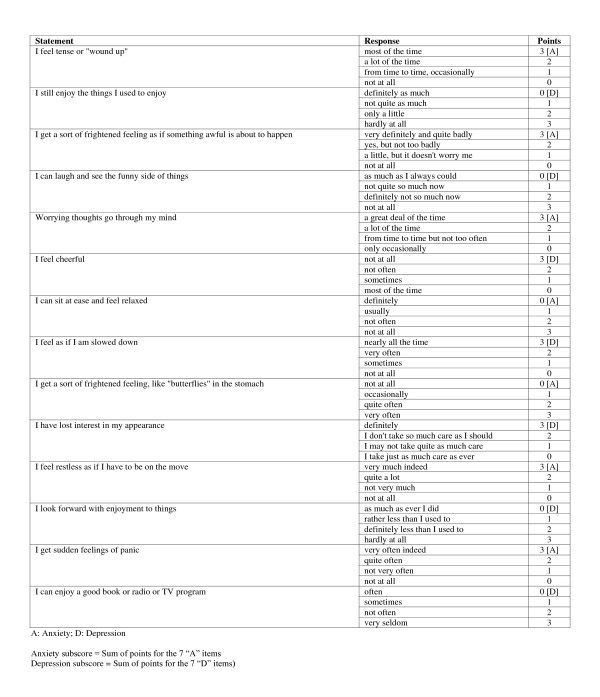
**A copy of the Hospital Anxiety and Depression (HAD) Scale**.

The participants' demographic and socioeconomic information were recorded on a special examination form and all participating subjects were asked whether they often experienced any of the following systemic symptoms: fatigue (without an obvious cause), loss of appetite, weight loss and depressive mood. The emotional status was further assessed for all subjects using the HAD Scale [20] (Figure 1). This scale is a self-assessment instrument that has been designed to detect anxiety and depression in medical outpatients [20]. The HAD scale consists of 14 statements (7 for anxiety, designated as "A" and 7 for depression, designated as "B"), with 4 possible responses for each statement. Each response is scored from 0-4 points. The minimum subscore for each category ("A" or "B") is zero and the maximum subscore is 21. According to the subscore of the HAD scale, the participants were divided into three groups, as recommended by the authors [20]; those who scored ≤ 7 were considered to be free of anxiety or depression, those who scored 8-10 were considered to have doubtful anxiety or depression and those whose subscore was ≥ 11 were considered to have anxiety or depression.

For each subject, full mouth periodontal examination was carried out by one of three examiners (AHT, MSA and KTA). The periodontal examination included measurement of Clinical Attachment Level (CAL) and the plaque index (PI) of Silness and Löe [21]. For measurement of CAL, each tooth was examined by "walking" the periodontal probe around the whole circumference of the tooth; third molars and remaining roots were excluded. CAL was measured at six sites per tooth (mesio-, mid-, and disto-buccal; mesio-, mid-, and disto-lingual/palatal). Inter-examiner reliability was calculated using alpha statistics with regard to probing depth and CAL on 16 quadrants. Diagnosis of CP and AP was based on CAL values and confirmed radiographically using intra-oral periapical and bitewing radiographs. Periodontitis was defined as the presence of attachment loss (CAL) > 2 mm on more than one tooth. For all participants bitewing radiographs were taken for posterior teeth and pariapical radiographs were taken for anterior teeth to detect the presence and pattern of alveolar bone loss and confirm (or exclude) the presence of periodontitis. To differentiate between CP and AP, the clinical findings including gingival condition, CAL, the severity and (to a lower extent) the pattern of bone loss, together with the subject's age were used as diagnostic criteria. When the subject had CAL > 2 mm around at least two teeth, one of which was a first molar, or when attachment loss was observed around first molars and/or incisors that exhibited bone loss at an early age (i.e. <45 years), especially were the characteristic arc-shaped defect(s) was/were detectable on radiographs, the case was diagnosed as AP. Inconsistence between the amount of plaque deposits and amount of periodontal destruction (whenever present), and positive family history further confirmed the diagnosis of AP. On the other hand, CP was diagnosed when CAL > 2 mm around at least two teeth, usually in older age groups (i.e. > 45 years). Young individuals with slight attachment and bone loss in whom plaque deposits were consistent with the amount of destruction were diagnosed as having CP. Cases where there was uncertainty in the diagnosis of AP or CP were not included in this study.

The investigated dental anomalies included dens invaginatus, dens evaginatus, congenitally missing and peg shaped lateral incisors. Congenitally missing teeth were recorded after verifying their congenital absence by the participants and their absence was confirmed using periapical radiographs. The presence of peg-shaped lateral incisors was noted and all teeth were examined both clinically and radiographically for the presence of dens evaginatus and dens invaginatus. Dens evaginatus cases were classified according to Oehlers (1957) [5].

### Statistical Analysis

All variables were entered into a personal computer, and the Statistical Package for Social Sciences (SPSS Version 11, Chicago, Illinois) software was used for data processing and analysis. Frequency distribution and cross-tabulation were produced. Mean values and standard deviation were calculated and Chi-square test was used. Differences were considered significant when p was < 0.05.

## Results

The Cronbach alpha coefficient was 0.94 for CAL, indicating excellent agreement between the examiners.

The mean CAL value for CP cases was 2.17 mm (± 1.53 SD), whereas the mean CAL value for AP cases was 2.76 mm (± 1.77 SD). The control subjects exhibited no attachment loss (mean CAL = 0 mm) and no radiographic evidence of alveolar bone loss.

### Sociodemographic Characteristics

As shown in Table 1, the highest proportion of CP patients were males, aged between 36 to 45 years, employed but with a low income and had up to high school education (i.e. ≤12 years). CP subjects and controls were significantly different with regard to age, occupation, place of residence and education. The highest percentage of AP subjects were young (≤25 years), were females, were unemployed, had a low income, lived in urban areas and had received up to high school education. Members of the AP group were significantly different from controls with respect to age, gender, occupation, income and education. When the CP and AP groups were compared, statistically significant differences were found between both groups with regard to age, gender, occupation, place of residence and education (Table 1).

**Table 1:** Socio-demographic characteristics of the study population

**Variables**		**CP**	**AP**	**Controls**	**P-value^a^**	**P-value^b^**	**P-value^c^**
					
		**No (%)**	**No (%)**	**No (%)**			
					
Age (Yrs)	≤ 25	10 (10)	31 (38.3)	63 (77.8)	< 0.0001	< 0.0001	< 0.0001
	26-35	24 (24)	25 (30.9)	13 (16)			
	36-45	34 (34)	24 (29.6)	5 (6.2)			
	≥ 46	32 (32)	1 (1.2)	-			
	Mean	39.9	29.8	22.2			
**Gender**	Female	45 (45)	57 (70.4)	35 (43.2)	0.464	< 0.0001	< 0.0001
	Male	55 (55)	24 (29.6)	46 (56.8)			
**Occupation**	Student	6 (6)	17 (21)	55 (67.9)	< 0.0001	< 0.0001	0.001
	Employed	58 (58)	28 (34.6)	21 (25.9)			
	Unemployed	36 (36)	36 (44.4)	5 (6.2)			
**Income (JOD)^d^**	≤350	84 (84)	70 (86.4)	59 (72.8)	0.067	0.032	0.65
	> 350	16 (16)	11 (13.6)	22 (27.2)			
**Residence**	Urban	50 (50)	53 (65.4)	63 (77.8)	< 0.0001	0.058	0.037
	Rural	50 (50)	28 (34.6)	18 (22.2)			
**Education**	**≤**High school	67 (67)	42 (51.9)	25 (30.9)	< 0.0001	0.005	0.038
					
	> High school	33 (33)	39 (48.1)	56 (69.1)			

^a ^CP vs. Controls; Chi-square test

^b ^AP vs. Controls; Chi-square test

^c ^CP vs. AP; Chi-square test

^d ^Monthly in Jordanian Dinars = $1.41

The control sample consisted of 81 systemically healthy, periodontitis-free Jordanian subjects; 45 males and 36 females, with an age range of 14-37 years, and a mean age of 22.2 years (± SD), in whom no clinical or radiographic evidence of attachment or bone loss was present at any site. The age of the controls was not restricted to 37 years, but it was virtually impossible to find periodontally healthy individuals aged 40 years or above.

### Systemic Manifestations

About 51% of AP patients reported that they often experienced one or more systemic symptoms (mostly fatigue and depressive mood), which they could not relate to disease or to external factors. A lower percentage of CP cases (36%) and controls (about 29%) reported the presence of such symptoms. The frequency of systemic manifestations was significantly greater in AP subjects than controls (*p *= 0.019). No significant differences were detected in the frequency of systemic symptoms between CP cases and controls (*p *= 0.7). However, marginally significant difference was observed between CP and AP cases (*p *= 0.059). Table 2 shows the differential distribution of the systemic manifestations reported by the study population. The most commonly reported systemic complaint by the 3 groups was fatigue, followed by depressive mood. Although depressive mood was more frequently reported by AP patients than the other 2 groups, the difference was not statistically significant.

**Table 2:** differential distribution of systemic manifestations

Systemic Manifestation	CP	AP	Controls	*P-values*		
	**No (%)**^**a**^	**No (%)**^**b**^	**No (%)**^**c**^	***CP vs. Control***	***AP vs. Control***	***CP vs. AP***

Fatigue	21 (21)	13 (16.0)	15 (18.5)	*0.405*	*0.851*	*0.229*
Loss of appetite	2 (2)	6 (7.4)	3 (3.7)	*1.000*	*0.508*	*0.289*
Weight loss	2 (2)	2 (2.5)	3 (3.7)	*1.000*	*1.000*	*1.000*
Depressive mood	5 (5)	9 (11.1)	3 (3.7)	*0.727*	*0.146*	*0.424*
***Total***	**36(36)**	**41(50.6)**	**24(28.9)**	***0.7***	***0.019***	***0.059***

^a ^Percentage out of a total of 100

^b ^Percentage out of a total of 81

^c ^Percentage out of a total of 81

### Anxiety and Depression using the HAD Scale

The anxiety and depression scores were summed independently to obtain an "anxiety score" and a "depression score" for each subject. Table 3 shows the numbers and percentages of individuals in each category of HAD scale scores. The group of highest percentage in this study scored 7 or less for both anxiety and depression. While almost equal proportions of AP (31%) and CP (32%) patients had doubtful anxiety, a much lower proportion of controls (14%) had doubtful anxiety. However, a higher percentage of patients with AP (31%) had definite anxiety than CP (21%) and controls (22%). Concerning depression scores, a higher percentage (26%) of AP cases had doubtful depression as well as definite depression (11%) than CP cases and controls. Table 3 also demonstrates that more AP patients (31%) had anxiety than depression (11%).

**Table 3:** HAD Scale for Anxiety and Depression among the study population

Variables		CP	AP	Controls	*P values*
			
		No (%)	No (%)	No (%)	
**Anxiety**	***≤ 7 ****(Not present)*	47 (47)	31 (38)	52 (64)	*0.49*^a^
	***8-10 ****(Doubtful)*	32 (32)	25 (31)	11 (14)	*0.039*^b^
	***≥11 ****(Definite)*	21 (21)	25 (31)	18 (22)	*0.74*^c^
	*Total*	100 (100)	81 (100)	81 (100)	
	Mean (± SD)	7.4 (± 3.9)	8.5 (± 3.4)	7 (± 3.8)	
					
**Depression**	***≤ 7 ****(Not present)*	68 (68)	51 (63)	67 (83)	*o.11*^d^
	***8-10 ****(Doubtful)*	23 (23)	21 (26)	10 (12)	*0.001*^e^
	***≥11 ****(Definite)*	9 (9)	9 (11)	4 (5)	*0.22*^f^
	*Total*	100 (100)	81 (100)	81 (100)	
	Mean (± SD)	5.8 (± 3.5)	6.8 (± 2.9)	4.8 (± 3.1)	

^a ^CP vs. Controls (Chi-square test)-Anxiety

^b ^AP vs. Controls (Chi-square test) -Anxiety

^c^CP vs. AP (Chi-square test) -Anxiety

^d ^CP vs. Controls (Chi-square test) -Depression

^e ^AP vs. Controls (Chi-square test) -Depression

^f ^CP vs. AP (Chi-square test) -Depression

The highest mean of anxiety and depression HAD scale scores (Table 3) was found in subjects with AP [8.5 (± 3.4) for anxiety and 6.8 (± 2.9) for depression], while the lowest scores were observed in the control group [7 (± 3.8) for anxiety and 4.8 (± 3.1) for depression]. A statistically significant difference was found when the anxiety (*p *= 0.039) and depression (*p *= 0.001) scores of AP patients were compared to controls. However, no significant differences were found in mean HAD scores by comparing CP and AP cases with controls (Table 3).

### Dental Anomalies

Dental anomalies were observed in 28 cases of the study population; in 15% of CP cases (15 subjects) and in 16% of AP cases (13 subjects), but were not observed in any of the control subjects (Table 4). All cases of dens invaginatus were observed uni- and bilaterally on the maxillary lateral incisors and were clinically and radiographically type I. All cases of dens evaginatus were small, cusp-like enlargements of the cingulum of maxillary lateral incisors and did not interfere with occlusion. Among AP cases (Table 5), 8 patients (9.9%) had dens invaginatus, one (1.2%) had dens evaginatus, 2 (2.46%) had bilateral peg-shaped lateral incisors and 2 patients (2.46%) had a congenitally missing upper lateral incisor. Among CP cases, 6 patients (6%), had dens invaginatus, 2 (2%) had dens evaginatus and 7 (7%) had unilateral congenitally missing teeth (2 lower second premolars and 5 maxillary lateral incisors). Both AP and CP were significantly more associated with dental anomalies than controls (*p *< 0.05), while the difference between the two periodontitis groups was not significant (*p *= 0.72). Furthermore, the disease groups did not significantly differ from controls or from each other when compared for each of the dental anomalies separately (*p *> 0.05).

**Table 4:** Dental Anomalies in Cases and Controls

Dental Anomalies	CP	AP	Controls	*P*-values^a^		
	No (%)	No (%)	No (%)	CP vs. Controls	AP vs. Controls	CP vs. AP
***Yes***	15 (15)	13 (16)	0 (0)	*0.004*	*0.003*	*0.72*
***No***	85 (85)	68 (84)	81 (100)			
***Total***	100 (100)	81 (100)	81 (100)			

^a ^Chi-square test

**Table 5:** Dental Anomalies in CP and AP.

Dental Anomaly	Site	CP	AP	Controls
		No (%)^a^	No (%)^b^	No (%)^c^
***Dens invaginatus***	Upper incisors	6 (6)	8 (9.9)	0 (0)
***Dens evaginatus***	Upper incisors	2 (2)	1 (1.2)	0 (0)
***Peg-shaped lateral incisors***	Upper lateral incisors	0 (0)	2 (2.46)	0 (0)
***Congenitally missing teeth***	Upper lateral incisors	7	2 (2.46)	0 (0)
	lower second premolars	2	0 (0)	0 (0)

^a ^Out of 1 total of 100

^b ^Out of a total of 81

^c ^Out of a total of 81

## Discussion

The distribution of chronic and aggressive periodontitis found in this study followed the general patterns reported by others [22-24]. The highest percentage of CP patients were older (> 35 years) than the highest percentage of AP patients (< 25 years). This confirms that AP is usually manifested earlier in life in susceptible individuals. While CP was distributed almost equally between males and females in this study, a greater proportion of AP patients were females. Surveys of periodontal conditions usually show that adult males are at a higher risk of developing CP than females [25]. This difference may be a reflection of better oral hygiene practices and more utilization of oral health care services among females rather than inherent differences between males and females regarding susceptibility to CP [26]. We found that the frequency of both forms of periodontitis was significantly lower in students as compared to employed and unemployed subjects. Socioeconomic level is a good marker of various risk factors for periodontitis such as oral hygiene, provision of dental care and behaviors. Previous studies have documented differences in periodontal health based on socioeconomic status (SES) factors, such as income and education, showing that lower SES was associated with increased risk to periodontitis [27]. However, education is currently believed to have a greater effect than income on the level of periodontitis in the population [28].

In this investigation certain systemic manifestations such as fatigue, loss of appetite, weight loss and depressive mood were investigated in relation to CP and AP. A significant proportion of patients diagnosed with AP reported that they experienced (one or more of these) systemic manifestations with the most frequently reported symptoms being fatigue and depressive mood. These findings are in accordance with those of Page et al. [2] who suggested that RPP (generalized AP) progresses in phases of activity and quiescence and that the active phase of RPP in a proportion of individuals involves systemic manifestations such as depression, general malaise, weight loss, and loss of appetite [2]. We have also observed that the frequency of these systemic manifestations is significantly greater in AP patients than in controls or CP patients (marginal significance). Evaluation of the anxiety and depression status of the participants in this study, using the HAD scale, demonstrated that subjects diagnosed with AP exhibited significantly more anxiety and depression, compared to CP patients and controls. It would be of interest to know how periodontitis (especially AP) is related to anxiety and depression. The bulk of literature has investigated the effect of psychological stress on periodontitis, but the effect of periodontitis on the psychological condition has not been the focus of much interest. The present study demonstrates mere association between periodontitis and both of anxiety and depression, and future longitudinal and multidisciplinary work is needed to shed light on this point. Furthermore, in the present study individuals with AP tended to score higher for anxiety than for depression. Anxiety in patients with AP may arise, in part, from their concern of losing teeth at a young age. It is also worth noting that most AP patients were unemployed, had a low income and had only (up to) high school education; unemployment, low income and education may give rise to instabilities in life and contribute to anxiety. However, it is not clear from the present results whether the presence of periodontitis and the poor prognosis of the dentition in this group of individuals have predisposed to anxiety and depression, or these psychological symptoms are true components of the disease (AP and possibly CP) as Page and colleagues [2] have suggested, and further studies are necessary to investigate this association.

Several dental anomalies were investigated in the present study including dens invaginatus, dens evaginatus, peg-shaped lateral incisors and congenitally missing lateral incisors. Interestingly, the dental anomalies investigated in this study were observed only in subjects with CP and AP, in contrast to controls where none of the dental anomalies investigated was present. Furthermore, the frequency of dens invaginatus observed among the AP (16%) and CP (15%) groups was significantly higher than that reported for the general population in Jordan (2.95%) [27]. It is believed that dental malformations are genetically determined because they are highly reproducible in shape, show predilection for some racial groups and often occur together [12]. The development of teeth is believed to be under strict genetic control, which determines the positions, numbers and shapes of different teeth [19]. Furthermore, dental anomalies, such as peg-shaped lateral incisors for example, are well documented components of numerous systemic diseases and syndromes, such as Down's syndrome [17], Witkop tooth and nail syndrome [28], Saethre-Chotzen syndrome [29], submucous cleft palate [30] and Hypohidrotic ectodermal dysplasia [31]. As the genetic basis for various dental anomalies is gradually being revealed [9], it is simultaneously becoming clearer that predisposition to various types of periodontitis is related to genetic polymorphisms in genes encoding certain cytokines and other components of the immune system, such as IL-1 [32] and IL-10 [33].

Therefore, it seems logical to postulate that certain dental anomalies may be components of AP and CP in some individuals resulting from specific, possibly related, genetic polymorphisms. This study, however, shows mere association and cannot confirm or exclude such an assumption. Genetic and large scale epidemiological studies, designed to investigate the association of AP and CP with individual dental anomalies are needed.

## Conclusions

It is concluded that the systemic manifestations of fatigue, depressive mood, loss of appetite and weight loss were strongly associated with AP. The dental anomalies dens invaginatus, dens evaginatus, peg-shaped and congenitally missing lateral incisors were found to be associated with aggressive and chronic periodontitis. The presence of these dental anomalies should encourage clinicians to perform thorough periodontal examination, and patients with aggressive periodontitis may be candidates for referral to professional psychological care.

## List of Abbreviations

**AP**: Aggressive Periodontitis; **CAL**: Clinical Attachment Level; **CEJ**: Cementoenamel Junction; **CP**: Chronic Periodontitis; **HAD scale**: Hospital Anxiety and Depression scale; **IL-1**: Interleukin 1; **IL-10**: Interleukin 10; **JOD**: Jordanian Dinar; **PI**: Plaque Index; **RPP**: Rapidly Progressive Periodontitis; **SES**: Socioeconomic Status.

## Competing interests

The authors declare that they have no competing interests.

## Authors' contributions

KTA put forward the research design, supervised and participated in data collection and wrote the manuscript. Both of AHT and MSA each carried out data collection and patient examination, and contributed to writing of the manuscript. JAK put forward the research design and participated in data analysis. YSK carried out the statistical analysis. All authors have read and approved the manuscript.
